# Load identification method for pepper harvesting drum based on dynamic chaotic characteristics of vibration-torque coupling

**DOI:** 10.1371/journal.pone.0352360

**Published:** 2026-06-29

**Authors:** Chen Wei, Jin Lei, Xinyan Qin, Yunshu Xiao, Zhi Wang, Shiguo Wang, Bin Li, Xiaohu Guo, Chengfu Wang

**Affiliations:** 1 College of Mechanical and Electrical Engineering, Shihezi University, Shihezi, China; 2 Mechanical Equipment Research Institute, Xinjiang Academy of Agricultural and Reclamation Science, Shihezi, China; 3 Shihezi Tianshan Machinery Manufacturing Co., Ltd, Shihezi, China; Federal University of Technology - Parana, BRAZIL

## Abstract

To improve the accuracy, robustness, and interpretability of load-state identification for pepper harvesting drums under complex field disturbances, this study proposes a load identification method based on the chaotic dynamics of vibration-torque coupling. Vibration signals reflect the structural dynamic response of the drum, whereas torque signals reflect load variations caused by crop-drum interaction. By modeling their nonlinear coupling, the proposed method captures load-sensitive dynamic evolution that is more discriminative than single-signal or conventional statistical features. This study aims to obtain reliable real-time drum load-state information to support adaptive adjustment of harvesting parameters, improve operational stability, and reduce fruit damage. A six-dimensional nonlinear coupled dynamical system was constructed by integrating Lorenz and Rössler models, explicitly representing drum load evolution through the cross-coupled nonlinear behavior of vibration and torque. A hybrid framework combining genetic algorithms and Gauss-Newton iteration was used for parameter identification, and the maximum Lyapunov exponent was extracted to quantify trajectory divergence under different load conditions, establishing a chaos-based and physically interpretable load characterization scheme. Based on Central Composite Design (CCD) experiments, the optimal operating parameters were determined as a drum rotational speed of 150 r/min and a forward speed of 0.42 m/s. Under these conditions, the picking rate reached 99.05%, the fruit damage rate was 2.35%, and the average load identification accuracy reached 90.47%. The AUC values for no-load, light-load, normal-load, and overload states were 0.992, 0.981, 1.000, and 0.947, respectively. Comparative experiments verified that the proposed method outperformed conventional load identification methods, and its successful implementation on embedded hardware demonstrated its applicability for on-machine real-time load identification. Overall, this method provides a robust and physically interpretable solution for pepper harvesting drum load identification and supports the optimization of pepper harvester operation.

## 1. Introduction

Pepper is a widely cultivated and versatile cash crop worldwide, and mechanized harvesting is widely used to improve harvesting efficiency. Among existing machines, the spring-tooth drum pepper harvester has been widely adopted because of its low fruit damage rate and high picking efficiency. During harvesting, the spring-tooth drum interacts with plants to detach fruits through brushing and facilitate subsequent separation [1]. As a key component, the drum load directly affects machine performance. Under field conditions, variations in plant architecture, fruit distribution density, uneven feeding, plant lodging, and soil-induced vibrations can cause rapid load fluctuations, intermittent overload events, reduced harvesting efficiency, increased fruit damage, and accelerated component wear [2–5]. Therefore, reliable drum load-state identification under real operating environments is essential for stable and efficient harvesting.

Condition monitoring of rotating machinery is important for improving reliability, reducing downtime, and extending service life [6]. For spring-tooth drum pepper harvesters, accurate identification of drum load states is particularly critical because field disturbances can directly affect harvesting quality, production costs, and operational stability [7,8]. Load-state information can also support adaptive adjustment of key parameters, such as drum rotational speed and forward speed, under changing crop conditions. With the development of machine learning, load-state identification for rotating components has increasingly relied on multi-source data fusion to improve recognition accuracy and robustness [9,10]. For example, Ma et al. [11] fused time-, frequency-, and time-frequency-domain features with principal component analysis and machine learning for load identification in combine harvester threshing and separating devices. Chen et al. [12] transformed vibration signals into time-frequency images and used multi-scale transformer fusion to improve long-period information extraction. These studies demonstrate the effectiveness of feature-based learning and deep architectures; however, their performance often depends on stable operating conditions and sufficiently representative training data.

Despite these advances, existing approaches still exhibit clear limitations in pepper drum load identification. First, many models primarily rely on statistical features or a single physical signal and lack an explicit representation of the drum system’s nonlinear dynamics. To address this gap, this study establishes a six-dimensional vibration-torque coupled chaotic model to describe the nonlinear dynamic response of the drum under different load states. Consequently, the theoretical basis remains weak, the intrinsic mechanisms linking load fluctuations to dynamic responses are difficult to interpret, and these models often struggle to handle operating condition variations in real field environments [13,14]. Second, although multi-source data fusion is widely adopted, it is frequently implemented as simple feature concatenation, neglecting the dynamic coupling between vibration and torque. To fill this gap, the proposed method introduces bidirectional coupling terms between the vibration-related Lorenz subsystem and the torque-related Rössler subsystem, so that the interaction between vibration and torque can be represented in a mechanism-consistent manner. During drum rotation, load-induced torque variations alter vibration patterns, while intensified vibration further affects torque fluctuations through mechanical transmission and structural interaction. This bidirectional vibration-torque modulation strengthens the separability of different load states, thereby improving identification accuracy, and also provides a mechanism-based explanation for how load changes are reflected in the measured signals. Without an explicit coupled dynamic model, critical information cannot be fully exploited, thereby limiting generalization and robustness across varieties, soil conditions, and harvesting densities [15,16]. More broadly, previous studies have demonstrated that variations in system parameters and operating conditions can be effectively reflected by measurable response characteristics, providing a theoretical and methodological basis for load-state identification using coupled dynamic signals [17–22]. Third, although deep learning can automatically learn representations, it depends heavily on large-scale labeled datasets. In pepper harvesting, the field environment is complex and data acquisition is costly, making it difficult to obtain sufficient labeled samples under diverse conditions. To reduce this dependence, this study extracts the maximum Lyapunov exponent as a load-sensitive chaotic feature and combines it with a multi-class SVM classifier, thereby improving identification performance under limited sample conditions. As a result, deep models are prone to overfitting and may show limited adaptability across varieties and field conditions, particularly in distinguishing subtle transitions (e.g., light load vs. normal load and normal load vs. overload). Moreover, many existing methods exhibit limited robustness to disturbances, hindering stable identification under the pronounced disturbances typical of agricultural operations. In particular, for practical decision-making, misclassification near critical boundaries may lead to inappropriate parameter adjustments, thereby increasing damage risk or reducing picking efficiency. To address the above challenges, this study focuses on drum load identification for spring-tooth pepper harvesters and develops an overall research framework that integrates multi-source vibration and torque information while emphasizing mechanism consistency and engineering applicability [23–25]. Relying on a dedicated harvesting test platform, multi-source signals are acquired under representative operating conditions to characterize differences in dynamic responses induced by drum load variations, thereby avoiding the limited applicability and poor interpretability associated with approaches that depend solely on a single signal or empirical statistical features. In addition, a systematic experimental design is adopted to organize operating parameter combinations, enhancing the coverage and transferability of the findings to practical harvesting scenarios. On this basis, comparative analyses and comprehensive validation are carried out to evaluate the stability and effectiveness of the proposed framework under complex disturbances [26–29]. Overall, the basic purpose of this work is to develop a physically interpretable and data-efficient method for identifying the load state of the pepper harvesting drum. The identified load information can provide a basis for adaptive regulation of drum rotational speed and forward speed, helping to maintain stable harvesting operation, reduce fruit damage, and prevent overload or blockage under complex field conditions. The main contributions of this paper are summarized as follows.

(1) A vibration-torque coupled chaotic dynamics model is established for pepper harvesting drums by integrating the Lorenz and Rössler systems into a six-dimensional nonlinear coupled dynamical model, so that load variation can be represented through mechanism consistent coupling behavior rather than single-signal descriptions.
(2) A chaos-based load characterization method is proposed by introducing the maximum Lyapunov exponent to quantify trajectory divergence and dynamic evolution under different loads, thereby enabling sensitive discrimination of subtle load transitions and improving physical interpretability beyond conventional statistical descriptors.
(3) An integrated load identification framework is constructed through CCD-based operating-parameter optimization, hybrid GA-Gauss-Newton parameter estimation, and multi-class load discrimination, and is validated by comparative experiments and on-machine tests for robust load identification, engineering applicability and stable harvesting operation with reduced pepper damage under field like disturbances.

## 2. Materials and methods

### 2.1 Equipment

**Fig 1:**
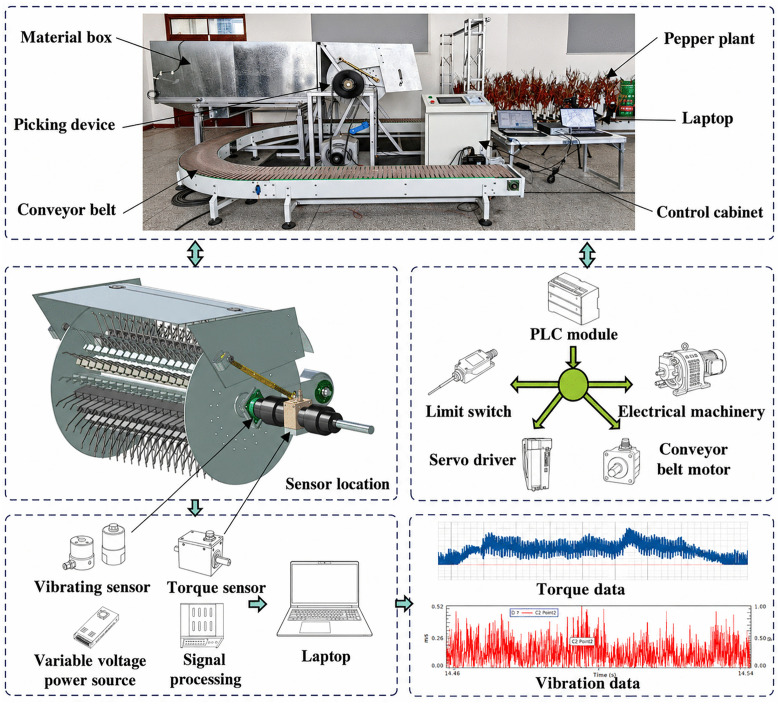
Structural diagram of the test platform equipment composition. The fig includes the chili pepper harvesting test rig schematic, the harvesting device schematic, PLC module, limit switch, servo driver, electrical machinery, conveyor belt motor, vibrating sensor, torque sensor, signal processing and variable voltage power source, laptop, torque data, and vibration data. Note: All components shown in the fig are existing laboratory devices. The fig was prepared by converting images or schematic materials of these devices into line drawings, and is used only to illustrate the composition of the experimental test platform. No third-party copyrighted material or intellectual property is involved.

The experimental platform mainly consists of a test device and a data acquisition system (Fig 1). The test device includes a conveying unit and a picking unit. The conveying unit adopts a U-shaped layout and is equipped with a slat chain conveyor. Multiple quick release horizontal clamps are uniformly mounted on the slat chain using bolts. The clamp type and mounting positions can be flexibly adjusted according to different row spacings, plant spacings, and cultivation patterns, thereby enabling changes in the feeding rate and plant density. The picking unit is centered on a spring tooth drum, which consists of two side plates and a tooth mounting plate installed between them. The spring teeth are mounted on the plate, and three adjacent rows of teeth are arranged in a staggered pattern. Both the tooth mounting plate and the teeth are removable, allowing convenient adjustment of the number of rows, the number of teeth per row, tooth spacing, and tooth type. The drum features a hollow structure to prevent pepper fruits from entering the drum and causing blockage during harvesting.

A DYN-200 torque sensor is installed at the front end of the drum and coupled to the main shaft to measure shaft torque and drum rotational speed in real time, from which power can be calculated. The measured torque variation reflects changes in shaft resistance caused by crop feeding, compression, friction, and material accumulation, and therefore provides a direct mechanical indication of drum load conditions. An INV9818 vibration sensor is mounted on the drum main shaft to acquire real-time vibration signals. These vibration signals reflect the mechanical behavior of the drum structure during pepper harvesting, including rotational excitation, crop-impact response, structural oscillation, and instability induced by nonuniform feeding. The drum is driven by a YCT-200 three phase asynchronous motor controlled by a frequency converter, allowing continuous speed regulation from 0 to 250 r/min. The main specifications of the torque and vibration sensors are summarized in Table 1.

**Table 1:** Main specifications of the torque and vibration sensors.

Torque Sensor		Vibration Sensor	
Model	DYN-200	Model	INV9818
Accuracy (%)	0.1	Sensitivity (mV/g)	142
Measurement Range (N·m)	0-2000	Frequency Range (kHz)	0.5-5
Pulse (Hz/r)	120	Operating Temperature (°C)	−20-100

### 2.2. Experiment

#### 2.2.1 Experimental factors.

The load on the spring tooth drum mainly comes from two sources. The first source is the load generated by the rotation of the spring-tooth drum itself, which is related to the drum rotational speed. The second source is the load generated by the interaction between the pepper plants and the drum during the pepper harvesting process. The magnitude of this load is related to the mass of peppers entering the drum per unit time. In actual fields, the fruit density of pepper plants varies due to differences in planting conditions and varieties. The feed rate is defined as the mass of pepper plants (including pepper fruits and impurities; the impurities are mainly branches, leaves, and stems of pepper plants) entering the drum per second, which is determined by the density of pepper plants, pepper yield, and forward speed in different fields. Therefore, in this study, drum rotational speed and forward speed were selected as the two experimental factors.

#### 2.2.2. Experimental indicators.

With reference to the methods and requirements for determining the indicators of spring-tooth drum pepper harvesters specified in the Industry Standard of the People’s Republic of China DG/T 114–2019: Program for Extension Appraisal of Agricultural Machinery [30], and combined with the design of the spring-tooth drum in this paper, pepper picking rate, damage rate, and feed rate were selected as the experimental indicators and are calculated using equations (1)-(3), as follows:


$$\begin{array}{c} Y=\frac{m}{m+m_{\text{1}}+m_{\text{2}}}\times \text{100}\% \end{array}$$ (1)

where Y  represents the pepper picking rate; m  is the mass of harvested peppers; $m_{\text{1}\;}$ is the mass of detached peppers, and $m_{\text{2}}\;$ is the mass of missed peppers.


$$\begin{array}{c} S=\frac{m_{\text{3}}}{m}\times \text{100}\% \end{array}$$ (2)

where S  represents the pepper damage rate; $m_{\text{3}}\;$ is the mass of damaged peppers. The feed rate is defined as:


$$\begin{array}{c} W=\frac{m+m_{\text{1}}+m_{\text{2}}+m_{\text{4}}}{T} \end{array}$$ (3)

where W  represents the feed rate; ${\text{m}}_{\text{4}}\;$ is the mass of impurities (mainly stems, branches, and leaves); and T  is the test time. All masses are measured in kilograms (kg), and T  is measured in seconds (s). Here, m, $m_{\text{1}}$, and $m_{\text{2}}$ denote pepper fruit masses, and $m_{\text{4}}$ is impurity mass.

#### 2.2.3 Single-factor experiments.

To evaluate the effects of drum rotational speed and forward speed on picking performance, single-factor experiments were conducted. The drum rotational speed and forward speed were selected because they correspond to the two dominant physical sources of drum load. Drum rotational speed determines the self-excited mechanical response of the rotating spring-tooth drum, including brushing intensity, impact frequency, rotational excitation, and structural vibration response. Forward speed determines the feeding intensity of pepper plants into the drum, thereby affecting crop-drum interaction, compression, frictional resistance, torque fluctuation, and material accumulation. According to the adjustable speed range of the test platform, the drum rotational speed can be regulated within 0–250 r/min. Considering the practical operating range of spring-tooth pepper harvesting drums and the need to cover low, medium, high, and boundary rotational-speed conditions, five drum rotational-speed levels were selected: 100, 130, 160, 190, and 220 r/min. These levels allow the effects of insufficient excitation, moderate harvesting operation, and excessive mechanical interaction on picking performance to be evaluated. For the forward speed, five levels were tested: 0.14, 0.28, 0.42, 0.56, and 0.70 m/s. These levels were selected to represent different feeding intensities under practical harvesting conditions, ranging from low-speed feeding to relatively high-speed harvesting. Each experimental level was repeated three times, and the average value was recorded for subsequent analysis.

#### 2.2.4 CCD experiment.

To further explore the interactive effects of the experimental factors and optimize parameter combinations, a CCD was adopted with forward speed and drum rotational speed as factors, and picking rate and damage rate as evaluation indicators. Factor levels and coding were set as follows. Forward speed (A): star points at 0.14 and 0.70 m/s, factorial points at 0.28 and 0.56 m/s, and the center point at 0.42 m/s. Drum rotational speed (C): star points at 100 and 220 r/min, factorial points at 130 and 190 r/min, and the center point at 160 r/min. The axial distance was 𝛼 = 1.414. The experimental structure comprised 11 runs, including 4 factorial points, 4 pivot points, and 3 replicated center points for error estimation.

### 2.3 Theoretical model

**Fig 2:**
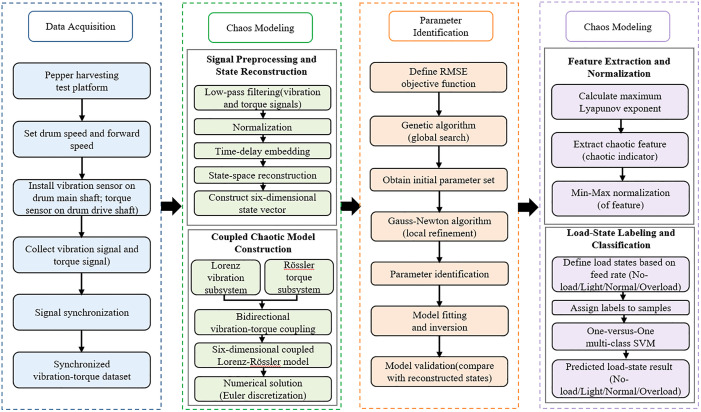
Workflow of the proposed vibration-torque coupled chaotic load identification framework, including data acquisition, chaos-based modeling, parameter identification, feature extraction, and load-state classification.

To improve the readability of the proposed methodology, the overall workflow of the vibration-torque coupled chaotic load identification framework is shown in Fig 2. The framework includes four stages: data acquisition, chaos-based modeling, parameter identification, and load-state classification. First, vibration and torque signals are collected, synchronized, filtered, and normalized. Then, time-delay embedding and state-space reconstruction are performed to build a six-dimensional coupled Lorenz-Rössler chaotic model. Next, the model parameters are identified using a hybrid genetic algorithm and Gauss-Newton optimization method, and the maximum Lyapunov exponent is extracted as a load-sensitive chaotic feature. Finally, the normalized feature is used for one-versus-one multi-class SVM classification to identify no-load, light-load, normal-load, and overload states. The code used to implement the proposed vibration-torque coupled chaotic load identification framework is available in S1 File.

#### 2.3.1. Theoretical basis.

In the load identification of the drum in pepper harvesters, the Lorenz-Rössler system was introduced not as an arbitrary mathematical model, but as a mechanism-consistent reduced-order nonlinear dynamic framework for describing the coupled vibration-torque response of the harvesting drum. The crop-drum interaction involves intermittent feeding, impact, compression, friction, material accumulation, and load fluctuation. These factors generate nonlinear excitation, time-varying resistance, irregular oscillation, and trajectory divergence, which are typical characteristics of chaotic dynamic systems. Therefore, the use of Lorenz and Rössler systems is justified by the nonlinear dynamic nature of the actual harvesting process. The core advantage lies in the high adaptability between its dynamic characteristics and the actual physical process [31–34]. The Lorenz subsystem is used to represent vibration-related nonlinear energy transfer and dissipation. In the harvesting drum, crop impact and rotational excitation continuously inject energy into the structure, while support damping and material damping dissipate energy. Under nonuniform feeding, this excitation-dissipation process becomes nonlinear and may lead to unstable vibration and trajectory divergence. Therefore, the Lorenz subsystem is physically consistent with the structural vibration mechanism of the drum. The Rössler subsystem is used to represent torque-related nonlinear feedback. Shaft torque is directly affected by crop feeding amount, crop compression, frictional resistance, and material accumulation. These load sources do not act as constant disturbances; instead, they produce time-varying resistance and feedback to the drive system, causing torque fluctuation and rotational instability. Therefore, the Rössler subsystem is physically consistent with the nonlinear feedback mechanism of drum torque evolution. Both systems are three-dimensional chaotic systems with a relatively simple structure, enabling the construction of a six-dimensional nonlinear dynamical model through cross-coupling terms. Recent neuro-computing studies have also demonstrated that artificial neural networks combined with meta-heuristic optimization algorithms can effectively approximate Lorenz differential equations and analyze nonlinear chaotic dynamics [35]. In this framework, the Lorenz subsystem describes vibration-related nonlinear energy transfer and dissipation, while the Rössler subsystem describes torque-related nonlinear feedback. Their coupling enables vibration-related and torque-related state variables to be represented in a unified dynamic space, which is suitable for characterizing real-time load variations caused by crop feeding and transient disturbances. This model can effectively reflect the dynamic interaction between vibration and torque, thereby providing a reliable dynamic framework for capturing multi scale coupled chaotic features and achieving accurate load identification.

#### 2.3.2. Model formulation and numerical implementation.

(1) Model Construction

The Lorenz system describes the chaotic behavior of atmospheric convection, and its three-dimensional state-space equations are expressed as follows [36]:


$$\begin{array}{c} \left\{ \begin{array}{cc} & {\dot{x}}_{\text{1}}=\sigma (x_{\text{2}}-x_{\text{1}})\\ & {\dot{x}}_{\text{2}}=x_{\text{1}}(r-x_{\text{3}})-x_{\text{2}} \end{array}\right.\; \end{array}$$ (4)

where $x_{\text{1}}$ is the drum vibration velocity characteristic, correlated with the first order integral of the bearing pedestal acceleration signal with drift compensation; $x_{\text{2}}$ is the vibration displacement gradient, reflecting the spatial distribution of drum unbalance; $x_{\text{3}}$ is the vibration energy dissipation term, correlated with the damping characteristics of the support structure; σ is the vibration mode coupling coefficient, determined by drum stiffness and damping ratio; r is the nonlinear excitation intensity, correlated with harvesting speed and pepper stalk stiffness; and b is the vibration attenuation factor, correlated with the material damping of the support structure.

The Rössler system describes the chaotic behavior of nonlinear feedback, and its three-dimensional state-space equations are expressed as follows [37]:


$$\begin{array}{c} \left\{ \begin{array}{cc} & {\dot{y}}_{\text{1}}=-y_{\text{2}}-y_{\text{3}}\\ & {\dot{y}}_{\text{2}}=y_{\text{1}}+ay_{\text{2}} \end{array}\right.\; \end{array}$$ (5)

where $y_{\text{1}}$ is the torque change rate, reflecting the inertia torque caused by sudden load changes; $y_{\text{2}}$ is the slow varying torque level, which is positively correlated with the harvesting amount; $y_{\text{3}}$ is the torque fluctuation amplitude, correlated with the stability of drum rotational speed; a is the damping coefficient of the drive system, determined by the lubrication state of the reducer; d is the constant load offset term, representing the system friction torque under no-load condition; and c is the torque feedback gain, correlated with the motor speed control parameters.

The measured vibration and torque signals were normalized prior to state mapping to obtain dimensionless variables and ensure consistency. From a physical perspective, the vibration-related state variables describe the mechanical response of the drum structure under rotational excitation, crop impact, structural deformation, and damping-related energy dissipation. The torque-related state variables describe the evolution of load resistance caused by intermittent crop feeding, crop compression, frictional resistance, material accumulation, and uneven crop-drum interaction. Therefore, the reconstructed state variables provide a physical bridge between the measured vibration-torque signals and the actual mechanical behavior and load conditions of the harvesting drum.

(2) Coupling and Numerical Solution

To characterize the bidirectional energy exchange between the drum vibration subsystem and the drive shaft torque subsystem, a linear cross injection mechanism is introduced. Let $\;x={\left[x_{\text{1}},x_{\text{2}},x_{\text{3}}\right]}^{\text{T}}\;$denote the Lorenz-state vector (vibration-related states) and $y={\left[y_{\text{1}},y_{\text{2}},y_{\text{3}}\right]}^{\text{T}}$ denote the Rössler state vector (torque-related states). The coupling inputs are constructed in a symmetric cross feedback form as:


$$\begin{array}{c} U_{x}=\psi M_{x}y{,\;U}_{y}=\psi M_{y}x \end{array}$$ (6)

where $U_{x}$ and $U_{y}$ are the coupling input vectors injected into the Lorenz and Rössler subsystems, respectively; and ψ is the coupling strength coefficient. In this study, the injection structure is selected as:


$$\begin{array}{c} M_{x}=[\begin{array}{ccc} \text{1} & \text{1} & \text{0}\\ \text{0} & \text{1} & \text{1}\\ \text{1} & \text{0} & \text{1} \end{array}],M_{y}=[\begin{array}{ccc} \text{1} & \text{1} & \text{0}\\ \text{0} & \text{1} & \text{1}\\ \text{1} & \text{0} & \text{1} \end{array}] \end{array}$$ (7)

where $M_{x}$ and $M_{y}$ are the coupling configuration matrices used to specify the cross-state injection structure between the Lorenz and Rössler subsystems.

Which yields the following six-dimensional coupled Lorenz-Rössler model:


$$\begin{array}{c} \left\{ \begin{array}{cc} & {\dot{x}}_{\text{1}}=\sigma (x_{\text{2}}-x_{\text{1}})+\psi (y_{\text{1}}+y_{\text{2}})\\ & {\dot{x}}_{\text{2}}=x_{\text{1}}(r-x_{\text{3}})-x_{\text{2}}+\psi (y_{\text{2}}+y_{\text{3}})\\ & {\dot{x}}_{\text{3}}=x_{\text{1}}x_{\text{2}}-bx_{\text{3}}+\psi (y_{\text{1}}+y_{\text{3}})\\ & {\dot{y}}_{\text{1}}=-y_{\text{2}}-y_{\text{3}}+\psi (x_{\text{1}}+x_{\text{2}})\\ & {\dot{y}}_{\text{2}}=y_{\text{1}}+ay_{\text{2}}+\psi (x_{\text{2}}+x_{\text{3}})\\ & {\dot{y}}_{\text{3}}=d+y_{\text{1}}y_{\text{3}}-cy_{\text{3}}+\psi (x_{\text{1}}+x_{\text{3}}) \end{array}\right.\; \end{array}$$ (8)

The terms injected into the Lorenz equation (e.g.,$\psi (y_{\text{1}}+y_{\text{2}})$, $\psi (y_{\text{2}}+y_{\text{3}})$, $\psi (y_{\text{1}}+y_{\text{3}})$) represent the feedback of torque-related states on the vibration dynamics, whereas the terms injected into the Rössler equation (e.g., $\psi (x_{\text{1}}+x_{\text{2}})$, $\psi (x_{\text{2}}+x_{\text{3}})$, $\psi (x_{\text{1}}+x_{\text{3}})$) represent the reverse effect of vibration states on torque evolution. This bidirectional linear coupling captures the mutual modulation between the two subsystems caused by mechanical transmission and structural interactions.

For numerical simulation, the explicit Euler method is adopted to discretize equation (8) with time step Δt according to standard numerical integration theory [38]:


$$\begin{array}{c} \left\{ \begin{array}{cccc} @lllll\overset{k+\text{1}}{\underset{\text{1}}{x}}=\overset{k}{\underset{\text{1}}{x}}+\Delta t\left[\sigma (\overset{k}{\underset{\text{2}}{x}}-\overset{k}{\underset{\text{1}}{x}})+\psi (\overset{k}{\underset{\text{1}}{y}}+\overset{k}{\underset{\text{2}}{y}})\right]\\ \overset{k+\text{1}}{\underset{\text{2}}{x}}=\overset{k}{\underset{\text{2}}{x}}+\Delta t\left[\overset{k}{\underset{\text{1}}{x}}(r-\overset{k}{\underset{\text{3}}{x}})-\overset{k}{\underset{\text{2}}{x}}+\psi (\overset{k}{\underset{\text{2}}{y}}+\overset{k}{\underset{\text{3}}{y}})\right]\\ \overset{k+\text{1}}{\underset{\text{3}}{x}}=\overset{k}{\underset{\text{3}}{x}}+\Delta t\left[\overset{k}{\underset{\text{1}}{x}}\overset{k}{\underset{\text{2}}{x}}-b\overset{k}{\underset{\text{3}}{x}}+\psi (\overset{k}{\underset{\text{1}}{y}}+\overset{k}{\underset{\text{3}}{y}})\right]\\ \overset{k+\text{1}}{\underset{\text{1}}{y}}=\overset{k}{\underset{\text{1}}{y}}+\Delta t\left[-\overset{k}{\underset{\text{2}}{y}}-\overset{k}{\underset{\text{3}}{y}}+\psi (\overset{k}{\underset{\text{1}}{x}}+\overset{k}{\underset{\text{2}}{x}})\right]\\ \overset{k+\text{1}}{\underset{\text{2}}{y}}=\overset{k}{\underset{\text{2}}{y}}+\Delta t\left[\overset{k}{\underset{\text{1}}{y}}+a\overset{k}{\underset{\text{2}}{y}}+\psi (\overset{k}{\underset{\text{2}}{x}}+\overset{k}{\underset{\text{3}}{x}})\right]\\ \overset{k+\text{1}}{\underset{\text{3}}{y}}=\overset{k}{\underset{\text{3}}{y}}+\Delta t\left[d+\overset{k}{\underset{\text{1}}{y}}\overset{k}{\underset{\text{3}}{y}}-c\overset{k}{\underset{\text{3}}{y}}+\psi (\overset{k}{\underset{\text{1}}{x}}+\overset{k}{\underset{\text{3}}{x}})\right] & & & \end{array}\right.\; \end{array}$$ (9)

where k=0,1,…,N−1 is the discrete time index, N is the number of sampling points, and the total acquisition duration is $T_{acq}=N\Delta t$. To analyze the local stability and chaotic characteristics of the coupled system, define the state vector $z=[x_{\text{1}},x_{\text{2}},x_{\text{3}},y_{\text{1}},y_{\text{2}},y_{\text{3}}{]}^{\text{T}}\;$and $\dot{z}=F(z)$. The Jacobian matrix is then defined as:


$$\begin{array}{c} J(z)=\frac{\partial F}{\partial z}={\left[\frac{\partial {\dot{z}}_{i}}{\partial z_{j}}\right]}_{\text{6}\times \text{6}} \end{array}$$ (10)

where z is the state vector of the coupled system, F(z) is the vector field of the coupled dynamical system, and J(z) is the Jacobian matrix of F(z) with respect to z.

For the coupled system $\dot{z}=F(z)$, the Jacobian $J(z)=\partial F/\partial z\in R^{\text{6}\times \text{6}}$ characterizes the instantaneous linear mapping from a perturbation in the state space to the corresponding perturbation in the velocity field. In other words, J(z(t)) describes how the local stretching and contraction rates are distributed across different state directions at time *t*.

To quantify the long-term sensitivity to initial conditions, the perturbation dynamics is governed by the variational equation $\dot{\delta z}=J(z(t))\delta z$. The maximum Lyapunov exponent is defined as the asymptotic average growth rate of infinitesimal perturbations and is commonly used to quantify the sensitivity of chaotic systems to initial conditions [39]:


$$\begin{array}{c} \lambda_{max}=\underset{t\to \infty }{lim}\;\frac{\text{1}}{t}ln\left(\frac{∥\delta z(t)∥}{∥\delta z(\text{0})∥}\right) \end{array}$$ (11)

where $\lambda_{max}$ is the maximum Lyapunov exponent, δz(0) is the initial infinitesimal perturbation vector, δz(t) is the perturbation vector at time *t*, and ∥·∥ is the norm of the perturbation vector.

In numerical implementation, direct integration of δz over long horizons suffer from vector alignment, where perturbation directions collapse toward the most unstable subspace, causing loss of numerical rank and biased estimates. Therefore, periodic re-orthonormalization (e.g., QR factorization or Gram-Schmidt) is applied to maintain an orthonormal perturbation basis and accumulate the logarithmic stretch factors. A positive $\lambda_{\text{max}}$ indicates that nearby trajectories diverge exponentially on average, implying strong local stretching on the attractor and serving as a quantitative indicator of chaotic instability. Moreover, variations in the maximum Lyapunov exponent under different load conditions reflect changes in trajectory divergence, excitation-dissipation balance, and vibration-torque coupling intensity. Therefore, the Lyapunov exponent improves accuracy by enhancing the discrimination of load states and improves interpretability by linking the classification feature to load-induced changes in drum dynamic stability [40–41].

#### 2.3.3 Signal processing.

(1) Filter Design

The drum rotational speed of the pepper harvester ranges from 0 to 250 r/min (0–4.17 Hz). However, vibration harmonics and structural resonance components may extend to higher frequencies. Therefore, a low-pass filter with a cutoff frequency of 100 Hz is applied to the vibration signal to retain the dominant mechanical features while suppressing high-frequency noise, thereby avoiding interference in the computation of the vibration energy dissipation related state (e.g., $x_{\text{3}}$).

Drive-shaft torque fluctuations are mainly induced by variations in harvesting amount and gear meshing excitations in the reducer. Accordingly, a low-pass filter with a cutoff frequency of 200 Hz is applied to the torque signal to cover its main dynamic range and attenuate high-frequency interference, improving the numerical stability and accuracy of the reconstructed torque related states (e.g., $y_{\text{1}}$ and $y_{\text{2}}$).

(2) Data Reconstruction

To achieve dimension matching from physical measurements to the six-dimensional coupled chaotic model, the measured vibration and torque signals are mapped to the state vector. This mapping transforms real-time vibration and torque measurements into a nonlinear state-space representation, so that load variations can be characterized by trajectory evolution, coupling behavior, and chaotic features rather than by instantaneous signal amplitudes alone. Since only two physical quantities (vibration and torque) are directly measured, a time delay reconstruction strategy is employed to augment the observation space and generate the additional variables required by the model structure. Specifically, four supplementary variables are derived from the two measured signals via time delay embedding, so that the reconstructed state set satisfies the dimensional requirement of the coupled Lorenz-Rössler system.

#### 2.3.4. Parameter estimation.

(1) Objective function

The objective function was defined based on the Root Mean Square Error (RMSE) between the model output of the pepper harvester’s harvesting drum vibration-torque coupling model and the measured signals:


$$\begin{array}{c} \text{F}(\theta )=\sqrt{\frac{\text{1}}{\text{6N}}\sum_{\text{k}=\text{1}}^{\text{N}}\;\sum_{\text{i}=\text{1}}^{\text{6}}\;(\overset{\text{k}}{\underset{\text{i}}{\text{z}}}-\overset{\text{k}}{\underset{\text{i}}{\hat{\text{z}}}}{)}^{\text{2}}} \end{array}$$ (12)

where θ=[σ,r,b,a,d,c] is the vector of parameters to be estimated, $\overset{k}{\underset{i}{z}}$ is the model-predicted value of the *i*-th state variable at the *k*-th sampling instant, $\hat{\overset{k}{\underset{i}{z}}}$ is the corresponding reconstructed state value obtained from the measured vibration and torque signals, and *N* is the length of the time series.

(2) Gauss-Newton least-squares iteration

A Gauss-Newton scheme is adopted to update parameters iteratively for nonlinear least-squares parameter estimation [42]:


$$\begin{array}{c} \theta^{m+\text{1}}=\theta^{m}-(J^{\text{T}}J{)}^{-\text{1}}J^{\text{T}}e \end{array}$$ (13)

where 𝑚 is the iteration index, 𝑒 is the residual vector, and 𝐽 is the Jacobian matrix of residuals with respect to parameters.

Step 1: Initialization

Initial parameter values are set according to classical chaotic system settings:


$$\begin{array}{c} \sigma =\text{10},r=\text{28},b=\text{8}/\text{3},a=\text{0}.\text{4},d=\text{1},c=\text{5}.\text{7} \end{array}$$ (14)

In this study, the coupling strength ψ was treated as a predefined coupling coefficient and was not included in the estimated parameter vector θ. A convergence threshold *ε* = 10^−6^ is used; the iteration terminates when the RMSE improvement satisfies  $\left|F^{\left(m+\text{1}\right)}-F^{\left(m\right)}\right|$＜ *ε.*

Step 2: Residual vector.

The residual vector is defined as the point wise difference between the model outputs and reconstructed states over all time steps and all six states, resulting in a vector of length 6𝑁:


$$\begin{array}{c} e={\left[\overset{\text{1}}{\underset{\text{1}}{x}}-\hat{\overset{\text{1}}{\underset{\text{1}}{x}}},\overset{\text{1}}{\underset{\text{2}}{x}}-\hat{\overset{\text{1}}{\underset{\text{2}}{x}}},...,\overset{N}{\underset{\text{3}}{y}}-\hat{\overset{N}{\underset{\text{3}}{y}}}\right]}^{\text{T}}\in R^{\text{6}N} \end{array}$$ (15)

Step 3: Jacobian matrix.

The Jacobian matrix J was constructed from the partial derivatives of the residuals with respect to the estimated parameters:


$$\begin{array}{c} J=\left[\begin{array}{c} \frac{\partial e}{\partial \theta_{\text{1}}},\frac{\partial e}{\partial \theta_{\text{2}}},\ldots ,\frac{\partial e}{\partial \theta_{p}} \end{array}\right] \end{array}$$ (16)

where *p = dim(θ)*. In numerical implementation, the sensitivities of the discretized model outputs to each parameter were calculated and assembled into J. The resulting residual Jacobian was then used in the Gauss-Newton update in Eq. (13). This simplified formulation retains the essential least-squares estimation procedure while avoiding lengthy intermediate sensitivity-propagation derivations.

(3) Hybrid optimization using genetic algorithm and Gauss-Newton

To improve the convergence reliability of nonlinear parameter estimation, a hybrid GA-Gauss-Newton strategy was adopted. The genetic algorithm first performed global optimization of the RMSE objective function to obtain a stable initial parameter vector. This result was then used as the initial value for Gauss-Newton local refinement, where the parameters were further updated using the residual Jacobian. This combined strategy reduces sensitivity to initial values and improves convergence stability compared with single-stage local optimization.

#### 2.3.5. Load identification performance of pepper harvesting drum load.

In this study, load identification refers to the process of determining the operating load state of the pepper harvesting drum from measured vibration and torque signals. The drum load is mainly generated by the interaction between the rotating spring teeth and the fed pepper plants, and it varies with crop density, feed rate, forward speed, and transient field disturbances. Since the instantaneous crop-drum interaction force is difficult to measure directly during field harvesting, load identification provides an indirect but practical way to evaluate the drum operating state. In this study, the maximum Lyapunov exponent was used as the primary quantitative chaos-based indicator for load-state identification. It quantifies the average exponential divergence rate of neighboring trajectories in the reconstructed vibration-torque coupled phase space. From a mechanical perspective, a larger maximum Lyapunov exponent indicates stronger trajectory divergence and greater dynamic instability of the drum, which is usually caused by nonuniform feeding, intermittent crop impact, frictional fluctuation, material accumulation, and unstable resistance torque. A smaller value indicates a more regular vibration-torque response and a more stable load state.

The maximum Lyapunov exponent was selected instead of binary chaos-detection metrics such as the 0–1 test because the purpose of this study is not only to determine whether chaotic behavior exists, but also to quantify load-dependent differences in nonlinear dynamic instability among no-load, light-load, normal-load, and overload states. The 0–1 test is mainly suitable for distinguishing chaotic and non-chaotic dynamics, whereas the maximum Lyapunov exponent provides a continuous and physically interpretable measure of trajectory divergence. Therefore, it is more suitable as the principal chaos-based feature for multi-state load classification in the proposed vibration-torque coupled framework.

Phase-space trajectories and attractor patterns were used as qualitative evidence of load-dependent dynamic evolution, whereas the normalized maximum Lyapunov exponent was used as the input feature for multi-class SVM classification. The performance of load identification was assessed using confusion matrices and Receiver Operating Characteristic (ROC) curves. A stratified sampling method was employed to divide the data into training and testing sets, ensuring that the proportion of each load category was consistent across both sets, thus avoiding potential biases in the model’s generalization performance caused by imbalanced sample distributions. A mapping relationship between the drum load conditions and various chaotic characteristics was subsequently established. The accuracy of load identification was then verified using the testing set samples.

The core input features in this study are chaotic characteristics, specifically the Lyapunov exponents, which characterize the degree of chaos in the system. It is noteworthy that the Lyapunov exponents exhibit significant differences under varying load conditions, making them suitable for distinguishing between different load states. Regarding feature data preprocessing, the Min-Max normalization method was applied to mitigate the impact of differences in feature magnitudes on the classification performance of the Support Vector Machine (SVM). The formula for Min-Max normalization is as follows:


$$\begin{array}{c} \overset{\text{norm}}{\underset{i}{L}}=\frac{L_{i}-\overset{\text{train}}{\underset{\text{min}}{L}}}{\overset{\text{train}}{\underset{\text{max}}{L}}-\overset{\text{train}}{\underset{\text{min}}{L}}}\times \text{2}-\text{1} \end{array}$$ (17)

where $L_{i}$ is the original Lyapunov-exponent-based feature value of the *i*-th sample, $\overset{\text{norm}}{\underset{i}{L}}$ is the normalized feature value, and $\overset{\text{train}}{\underset{\text{min}}{L}}$ and $\overset{\text{train}}{\underset{\text{max}}{L}}$ denote the minimum and maximum Lyapunov feature values in the training set, respectively.

A one-versus-one multi-class SVM classifier was adopted for load classification, as it constructs binary classifiers for each pair of categories, making it well suited for distinguishing between multiple load classes in a fine-grained manner. Given the nonlinear mapping between chaotic characteristics and load states, the Radial Basis Function (RBF) kernel was chosen to capture the nonlinear classification boundaries in the high dimensional feature space.

To evaluate model performance, metrics such as classification accuracy, confusion matrices, and ROC curves were generated. The confusion matrix provides insights into the model’s ability to correctly classify each load state, highlighting the true positive, false positive, true negative, and false negative rates across different load states. Additionally, the ROC curve was used to visually demonstrate the performance of the load identification model at various thresholds, plotting the relationship between the true positive rate and the false positive rate. The curve helps to assess the trade-off between sensitivity and specificity, facilitating the selection of an optimal threshold for classification. Furthermore, to ensure robust performance on imbalanced datasets, the model’s performance was further validated using metrics such as F1 score, precision, and recall. These metrics provide a more comprehensive evaluation of the model’s ability to correctly identify load states, especially in cases where class distribution is skewed.

#### 2.3.6 Multi-class SVM strategies: one-versus-one and one-versus-rest.

To further clarify how the chaos-based feature parameters (e.g., Lyapunov exponents) are mapped to four load states and why the classifier can distinguish subtle boundary cases, this subsection introduces two standard multi-class SVM schemes: one-versus-one and one-versus-rest. This addition is motivated by the observation that adjacent load conditions may exhibit partial feature overlap (particularly overload versus normal load), which increases classification difficulty; therefore, the chosen multi-class decomposition affects how well the model separates feature parameter distributions near critical boundaries.

(1) Binary SVM decision function and kernel

Given training samples $\{({𝐪}_{i},{\text{𝓁}}_{i})\overset{n_{s}}{\underset{i=\text{1}}{\}}}$, where ${𝐪}_{i}\in R^{m}$ is the SVM input feature vector and ${\text{𝓁}}_{i}\in${−1, + 1} is the binary class label, the soft-margin SVM solves [43]:


$$\underset{𝐰,b_{\text{0}},\xi }{min}\;\frac{\text{1}}{\text{2}}∥𝐰{∥}^{\text{2}}+C_{\text{svm}}\sum_{i=\text{1}}^{n_{s}}\;\xi_{i},\text{s}.\text{t}.{\text{𝓁}}_{i}\left({𝐰}^{\text{T}}\varphi ({𝐪}_{i})+b_{\text{0}}\right)\geq \text{1}-\xi_{i},\xi_{i}\geq \text{0},i=\text{1},\text{2},\ldots ,n_{\text{s}}$$ (18)

where *w* is the normal vector of the separating hyperplane, $b_{\text{0}}$ is the SVM bias term, $\xi_{i}$ is the slack variable, $n_{\text{s}}$ is the number of training samples, $C_{\text{svm}}$ is the penalty factor, and φ(·) is a nonlinear feature mapping.


$$f(𝐪)=\text{sign}\left(\sum_{i=\text{1}}^{n_{s}}\;\alpha_{i}{\text{𝓁}}_{i}K({𝐪}_{i},𝐪)+b_{\text{0}}\right)$$ (19)

where $\alpha_{i}$ is the Lagrange multiplier, $K(q_{i},q)$ is the kernel function, and sign(·) is the sign function.

Considering the nonlinear mapping between chaotic characteristics and load states, the radial basis function (RBF) kernel is adopted to capture nonlinear classification boundaries according to kernel-based SVM theory [44]:


$$\begin{array}{c} K(𝐪,{𝐪}^{\prime })=\text{exp}\left(-\gamma ∥𝐪-{𝐪}^{\prime }{∥}^{\text{2}}\right) \end{array}$$ (20)

where *γ* controls the kernel width and thus the boundary complexity.

(2) Feature scaling (used to separate feature-parameter magnitudes)

Because chaotic features values can differ significantly in magnitude across load conditions, feature scaling is required to avoid dominance by large magnitude dimensions and to improve SVM margin estimation. In this work, Min-Max normalization is applied before SVM training.

(3) One-versus-one multi-class strategy

For $K_{cls}$ load classes (here $K_{\text{cls}}$= 4: no-load, light load, normal load, overload), one-versus-one trains a binary classifier for every pair of classes, requiring:


NOvO=((Kcls2))=Kcls(Kcls−1)2 (21)

Thus, NOvO=((42))=6 classifiers are trained. Final prediction is obtained by majority voting among pairwise decisions. This scheme is particularly suitable for fine grained discrimination because each classifier focuses only on the boundary between two classes, which can reduce boundary distortion caused by pooling “all remaining classes” into a heterogeneous negative set.

(4) One-versus-rest multi-class strategy

one-versus-rest trains one classifier per class, separating class k from all other classes:


$$\begin{array}{c} {\text{N}}_{\text{OvR}}=K_{\text{cls}} \end{array}$$ (22)

At inference, the predicted class is typically selected by the maximum decision score:


$$\begin{array}{c} \hat{k}=arg\underset{c\in \{\text{1},\ldots ,K_{\text{cls}}\}}{max}\;g_{c}(q) \end{array}$$ (23)

where $\hat{\text{𝓁}}$ is the predicted class label, ccc is the class index, $g_{c}(q)$ is the real-valued output of the ccc-th One-versus-rest classifier, and $K_{\text{cls}}$ is the number of load classes.

one-versus-rest reduces the number of models and can be computationally cheaper, but the negative set (“rest”) is often complex and imbalanced, which may degrade separability for adjacent loads when feature overlap exists.

## 3. Results

### 3.1. Experimental results of single-factor and CCD analysis

#### 3.1.1. Single-factor experiment results.

To investigate the influence of drum rotational speed on the picking performance of the spring-tooth drum, five drum rotational speed levels (100, 130, 160, 190, and 220 r/min) were tested. Each group was repeated three times, and the average value was calculated. The results showed that as the drum rotational speed increased, the damage rate increased from 1.66% to 8.10%, while the picking rate first increased and then decreased. The picking rate reached a peak of 99.52% at 160 r/min. Beyond this drum rotational speed, the picking rate decreased because the excessive brushing speed of the spring teeth caused peppers to be thrown off. Therefore, 160 r/min was identified as the optimal drum rotational speed in the single-factor tests.

With the drum rotational speed fixed at 160 r/min, five forward speed levels (0.14, 0.28, 0.42, 0.56, and 0.70 m/s) were tested. The results indicated that as the forward speed increased, the damage rate first decreased and then increased, reaching the minimum of 2.16% at 0.42 m/s. The picking rate decreased from 99.67% to 98.65%. When the speed exceeded 0.42 m/s, the number of missed pickings increased due to the reduced brushing frequency of the spring teeth.

#### 3.1.2. CCD experimental design and results.

Following the single-factor experiments, the CCD was used to further investigate the combined effects of forward speed (A) and drum rotational speed (C) on picking performance. Table 2 shows the results of the CCD experiments, which include both factorial points and star points designed to explore the parameter space more comprehensively.

**Table 2:** Results of the CCD experiment for pepper harvesting.

Test No.	*A* (m/s)	*C* (r/min)	Type	Picking rate *Y* (%)	Damage rate *S* (%)
1	0.28	130	Factorial point	98.80	2.90
2	0.28	190	Factorial point	98.50	4.80
3	0.56	130	Factorial point	98.20	2.70
4	0.56	190	Factorial point	97.90	5.20
5	0.14	160	Star point	99.60	3.45
6	0.70	160	Star point	98.55	3.75
7	0.42	100	Star point	98.95	1.70
8	0.42	220	Star point	99.30	7.80
9	0.42	160	Center point	99.45	2.80
10	0.42	160	Center point	99.40	2.75
11	0.42	160	Center point	99.50	2.85

The CCD experiment results indicated the following: At A = 0.28 m/s and C = 130 r/min, the picking rate was 98.80%, with a damage rate of 2.9%. At A = 0.42 m/s and C = 160 r/min (center point), the picking rate was 99.45%, with a damage rate of 2.80%. At A = 0.42 m/s and C = 220 r/min, the picking rate was 99.30%, but the damage rate increased to 7.80%, suggesting that excessive drum rotational speed leads to more damage despite a high picking rate.

These results complement the findings from the single-factor experiments and confirm that there is an optimal range for both forward speed and drum rotational speed that maximizes the picking rate while minimizing the damage rate. The results from the CCD experiments provide a more refined understanding of how these two factors interact.

#### 3.1.3. CCD regression models and analysis of variance.

The picking-rate model was extremely significant (P < 0.0001), and the quadratic term of factor C (C²) had the most significant effect (F = 71.25). The fitted regression equation is:


$$\begin{array}{c} Y\;=\;\text{9}\text{9}.\text{4}\text{5}-\;\text{0}.\text{5}\text{2}A\;+\;\text{0}.\text{3}\text{8}C\;-\;\text{0}.\text{2}\text{1}AC\;-\;\text{0}.\text{6}\text{8}A^{\text{2}}\;-\;\text{0}.\text{8}\text{7}C^{\text{2}} \end{array}$$ (24)

The damage-rate model was extremely significant (P < 0.0001), and the effect of factor *C* was much greater than that of factor *A*. The fitted regression equation is:


$$\begin{array}{c} S\;=\;\text{2}.\text{8}\text{0}-\;\text{0}.\text{4}\text{3}A\;+\;\text{1}.\text{2}\text{6}C\;+\;\text{0}.\text{3}\text{2}AC\;+\;\text{0}.\text{7}\text{5}A^{\text{2}}\;+\;\text{1}.\text{5}\text{2}C^{\text{2}} \end{array}$$ (25)

where *A* and *C* are coded variables of forward speed and drum rotational speed, respectively.

#### 3.1.4 Response surface and interaction analysis.

**Fig 3:**
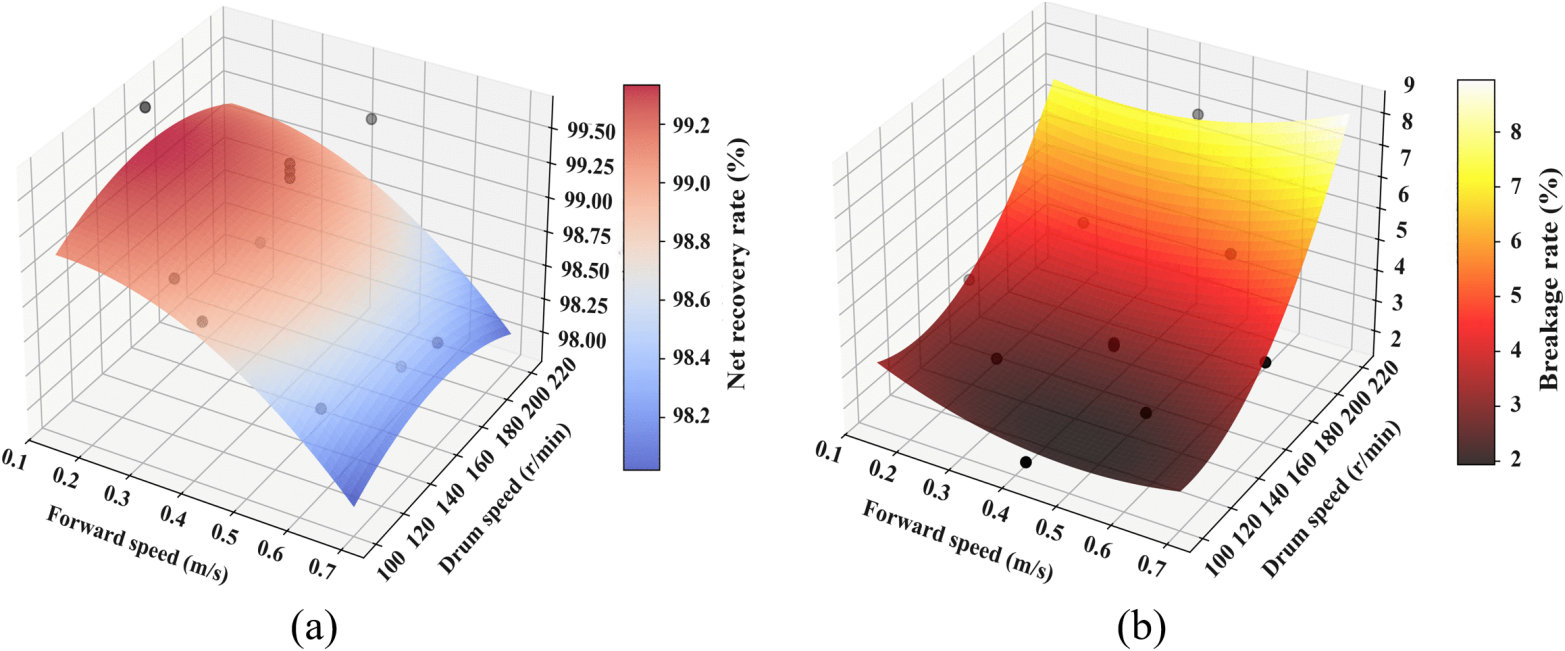
Response surface plots showing the effects of forward speed A and drum rotational speed C on pepper harvesting performance: (a) picking rate Y; (b) damage rate S. Axis labels and units were added as forward speed A (m/s), drum rotational speed C (r/min), picking rate Y (%), and damage rate S (%). The color bars were labeled to indicate the predicted response values.

Based on the fitted regression models in equations (27) and (28), response surface plots were generated to visualize the combined effects of *A* and *C* on the picking rate (*Y*) and damage rate (*S*). The response surfaces provide an intuitive interpretation of factor effects and support the identification of parameter regions that achieve a high picking rate while limiting damage (Fig 3).

From the response surfaces, a feasible operating region satisfying 𝑌 ≥ 98.5% and 𝑆 ≤ 3.5% was identified. This region forms the basis for the multi-objective optimization and verification presented in the following section.

### 3.2. Multi-objective optimization and verification

Based on the constraint conditions: *Y* ≥ 98.5%, *S* ≤ 3.5%, *A*∈ [0.14, 0.70] m/s, and *C*∈ [100, 220] r/min, a multi-objective optimization was performed to maximize the picking rate (*Y*) and minimize the damage rate (*S*) within the feasible operating region. The single objective extrema indicated that the maximum *Y* was obtained at A = 0.38 m/s and C = 152 r/min (Y = 99.6%). When S reached the minimum value, the parameters were A = 0.45 m/s and C = 130 r/min (S = 2.5%, while *Y* = 98.2% was slightly lower than the constraint). Weighted balanced solution: The optimal parameters were determined as A = 0.42 m/s and C = 150 r/min, corresponding to Y = 99.2% and S = 3.2%, which satisfied both objectives. Verification tests were carried out under the optimized parameters. The average values of three parallel tests were Y = 99.05% and S = 2.35%, demonstrating that the optimized parameters achieve high picking performance with low damage under practical operating conditions.

### 3.3. Load definition and dataset labeling

Based on the recommended operating parameters, the drum load was classified into four categories according to the feed rate *W* (corresponding to the density of pepper plants and fruits): No-load: W = 0 kg/s; Light load: 0 < *W* ≤ 0.570 kg/s; Normal load: 0.570 < *W* ≤ 1.92 kg/s; Overload: *W* > 1.92 kg/s. These feed-rate-defined labels were used as the ground truth for quantitatively evaluating the discrimination capability of the maximum-Lyapunov-exponent-based chaotic feature. The predicted load states were compared with the ground-truth labels using classification accuracy, confusion matrix, and ROC-AUC values. Using these feed-rate thresholds, each operating segment was assigned a corresponding load label, and the labeled samples were subsequently used as ground truth for feature extraction and supervised classification. The raw experimental data used for load-state labeling, feature extraction, and supervised classification are available in S2 File.

### 3.4. State-space reconstruction results

**Fig 4:**
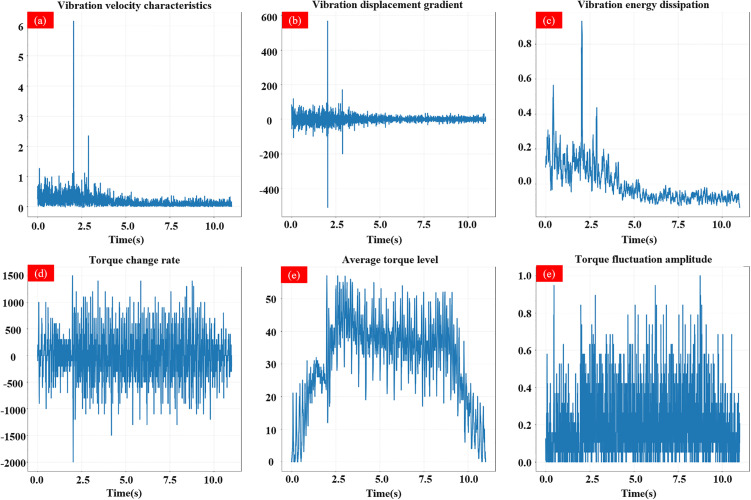
State variable trajectories of the reconstructed coupled system. Time-series response of key system variables: (a) vibration velocity characteristics; (b) vibration displacement gradient; (c) vibration energy dissipation; (d) torque change rate; (e) average torque level; (f) torque fluctuation amplitude. Time is in seconds; other axes in appropriate units.

An illustrative example of the reconstructed trajectories of the six state variables $\;\left[x_{\text{1}},x_{\text{2}},x_{\text{3}},y_{\text{1}},y_{\text{2}},y_{\text{3}}\right]$, obtained via the time-delay reconstruction described in Section 2.3.3. The reconstructed variables provide a six-dimensional state representation that is consistent with the coupled Lorenz-Rössler model structure, enabling subsequent parameter estimation and chaos related feature evaluation within a unified dynamical framework (Fig 4).

### 3.5. Parameter identification of the coupled model

#### 3.5.1. Genetic algorithm-based parameter optimization.

Following the reconstruction process, a genetic algorithm was employed to perform a global search and obtain a high-quality initial parameter set for subsequent Gauss-Newton refinement. For the experimental dataset considered, the genetic algorithm converged after 15 iterations, yielding an RMSE of 182.74 and the optimized parameter vector shown in equation (29).


$$\begin{array}{c} \sigma =\text{18}.\text{55},r=\text{23}.\text{33},b=\text{1}.\text{42},a=\text{0}.\text{51},d=\text{4}.\text{41},c=\text{7}.\text{28} \end{array}$$ (26)

This result indicates that the global search stage effectively avoided unstable initialization and provided a suitable starting point for local optimization. After Gauss-Newton refinement, the identified parameters could better fit the reconstructed vibration-torque trajectories and characterize different drum states under varying loads.

#### 3.5.2. Inversion performance verification.

**Fig 5:**
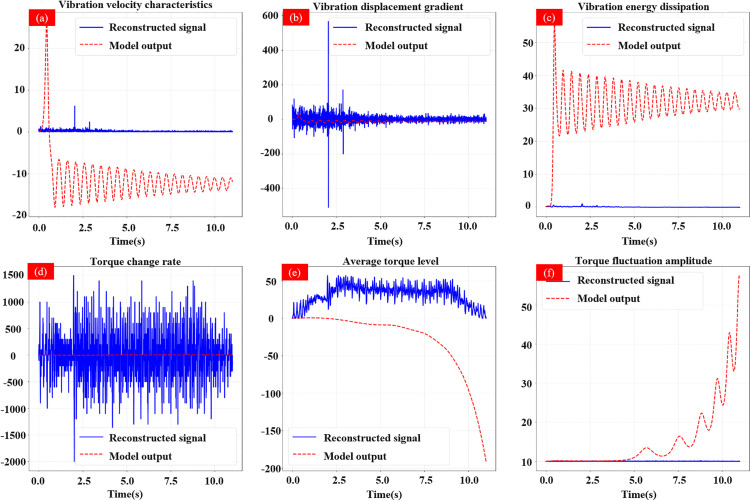
Comparison of reconstructed states and model outputs after parameter inversion. Time-series comparisons of vibration velocity, displacement gradient, energy dissipation, torque change rate, average torque, and torque fluctuation. Blue solid lines denote reconstructed signals, red dashed lines denote model outputs. The model captures the main temporal patterns despite local deviations.

After parameter optimization, the model outputs reproduce the major dynamic evolution trends of the reconstructed trajectories, although local deviations remain under certain operating conditions due to strong nonlinear disturbances and stochastic field effects. Overall, the model reproduces the main temporal patterns of the reconstructed trajectories, indicating that the identified parameters capture the dominant coupled dynamics between vibration and torque (Fig 5).

The reconstructed state trajectories capture the essential dynamics of the drum under all representative load conditions. The deviations between the model predicted states and the reconstructed measurements remain small, confirming the robustness and reliability of the parameter identification procedure. Across all load conditions, the reconstruction errors are consistently low, illustrating the accuracy of the identified parameters and the effectiveness of the inversion method.

### 3.6 Phase portraits and Lyapunov-based load characterization

**Fig 6:**
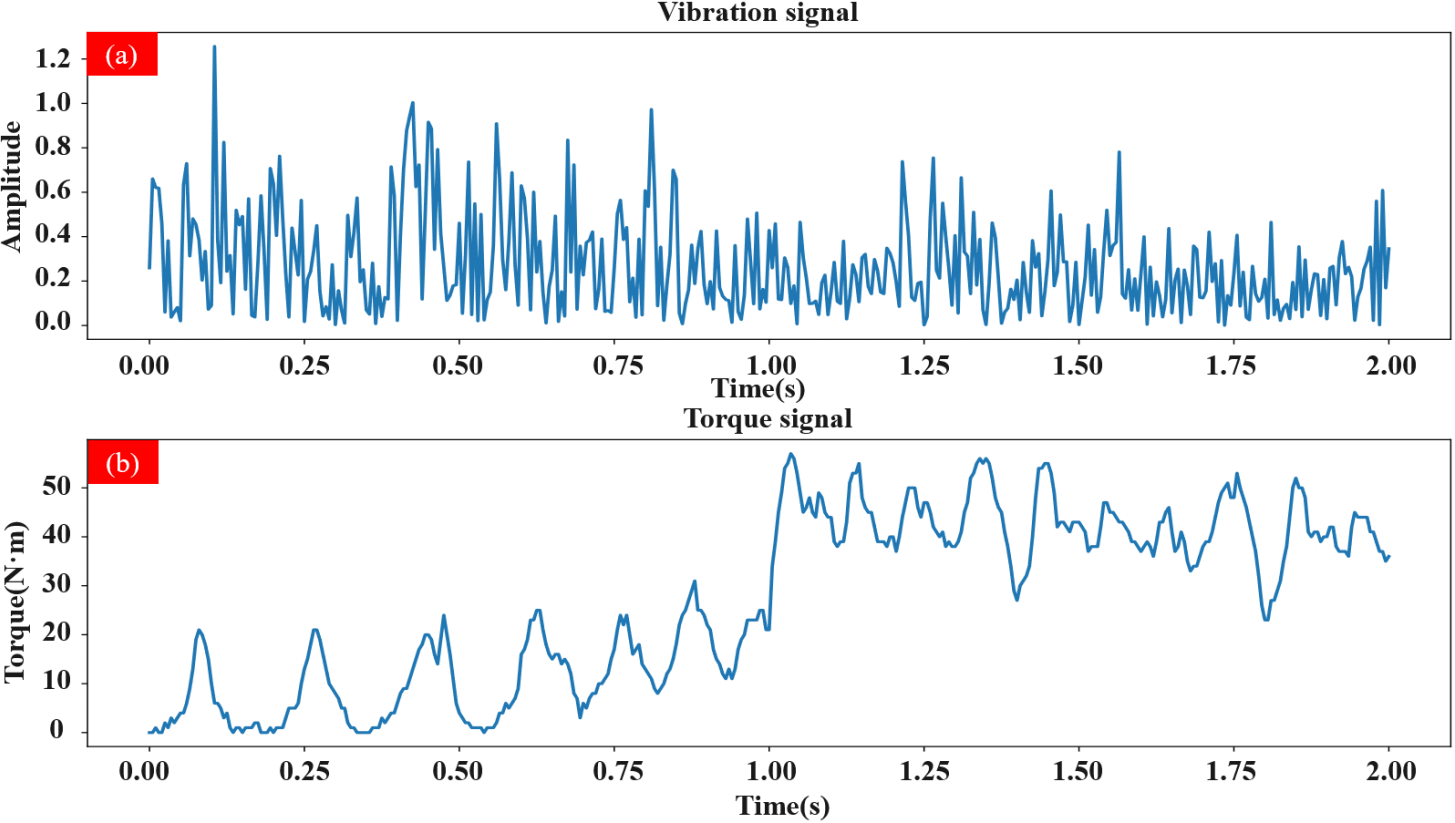
Vibration and torque signal diagram from no-load to normal load. (a) vibration amplitude (b) torque response over time. The no-load, transition, and normal-load regions are annotated in the fig. The signals reveal distinct dynamic patterns across load regimes, capturing the system’s dominant temporal behavior and providing a qualitative basis for Lyapunov-exponent analysis.

With the coupled-model trajectories validated in Section 3.5 (Fig 5), this sub-section examines how the phase-space structure and Lyapunov-based chaotic features evolve with drum load, thereby establishing dynamical indicators for load characterization. It shows the time-domain vibration and torque signals during a transition from no-load to loaded operation (Fig 6). Distinct attractor patterns are observed across different load regimes, indicating a clear dynamical shift as the drum processes varying feed rates and providing a qualitative basis for subsequent Lyapunov-exponent quantification.

**Fig 7:**
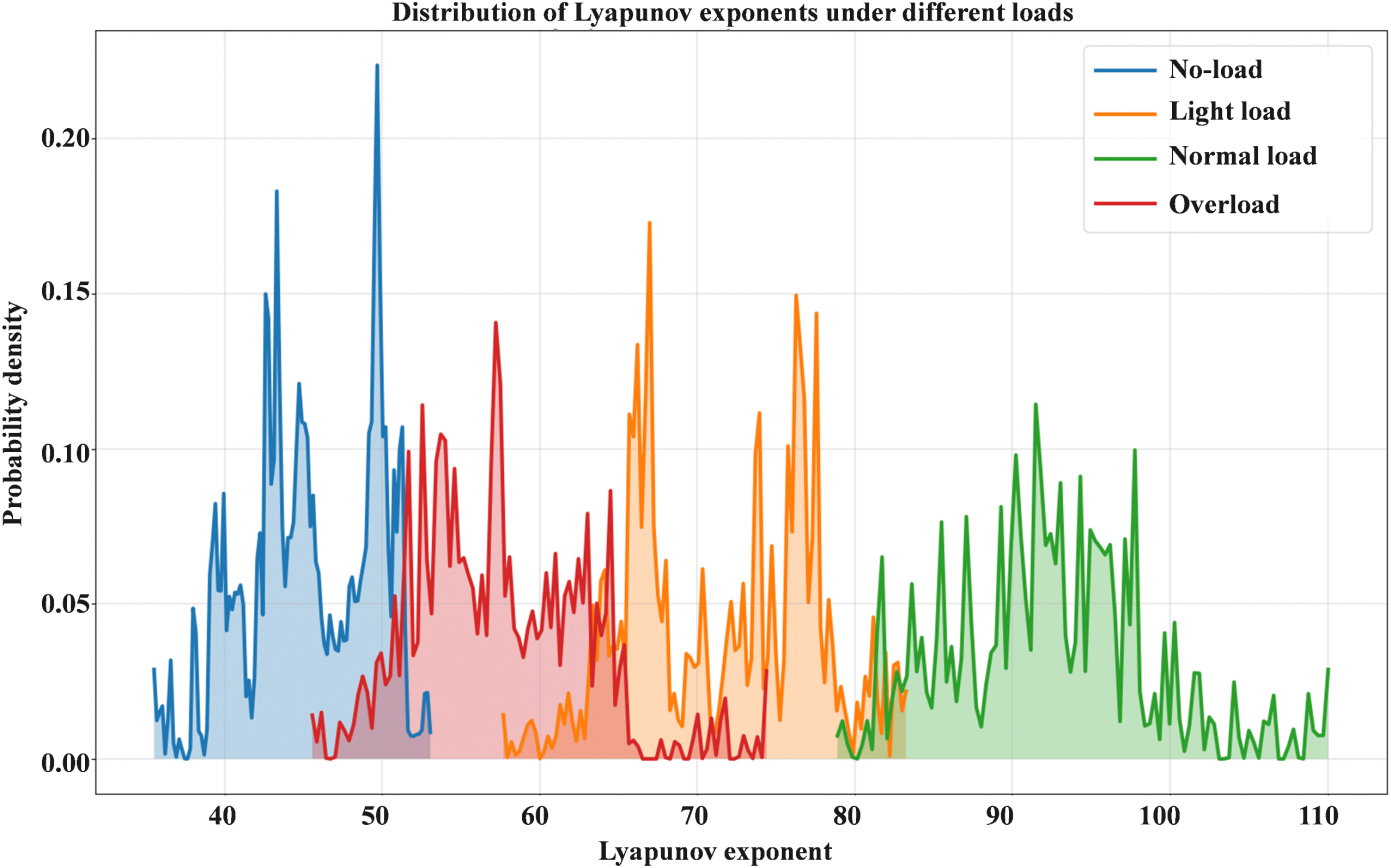
Lyapunov exponent distributions under four load conditions. The load-state labels were added as no-load, light load, normal load, and overload. The y-axis was labeled as maximum Lyapunov exponent, and the legend/color description was clarified to distinguish the four load states.

This further illustrates the Lyapunov exponent distribution of the coupled system under four load conditions (Fig 7). The distributions of the maximum Lyapunov exponent under no-load, light-load, normal-load, and overload conditions showed distinguishable intervals, confirming that this chaotic feature contains effective load-state information. However, the exponent did not vary strictly monotonically with load level because of nonlinear vibration-torque coupling, stochastic feeding, and field disturbances. Therefore, the maximum Lyapunov exponent was used as a load-sensitive classifier input rather than as a fixed threshold for real-time identification.

### 3.7. Load identification results and multi-class strategy analysis

#### 3.7.1. Confusion matrix results.

**Fig 8:**
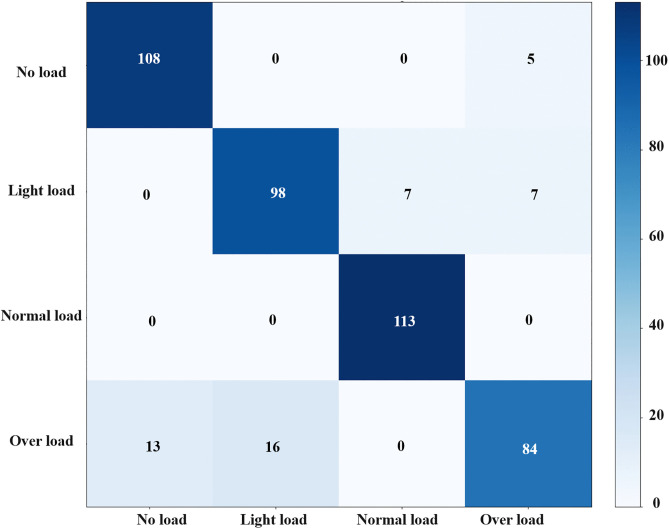
Confusion matrix for load state classification, showing high agreement between predicted and actual states (average accuracy 90.47%) and demonstrating the effectiveness of the maximum-Lyapunov-exponent feature with multi-class SVM.

The confusion matrix showed that the average identification accuracy reached 90.47%, and the normalized maximum-Lyapunov-exponent feature combined with multi-class SVM effectively distinguished the four load states, indicating strong recognition ability (Fig 8). As shown in the matrix, the majority of samples are correctly classified, with most values concentrated on the main diagonal, reflecting a high level of agreement between the predicted and actual labels. Misclassifications occur only in a limited number of cases and are mainly distributed among categories with similar load characteristics, suggesting that the model is sensitive to subtle differences between load states. Overall, these results confirm that the proposed model exhibits good discriminative performance, stability, and practical applicability for load category prediction.

#### 3.7.2 ROC-AUC results.

**Fig 9:**
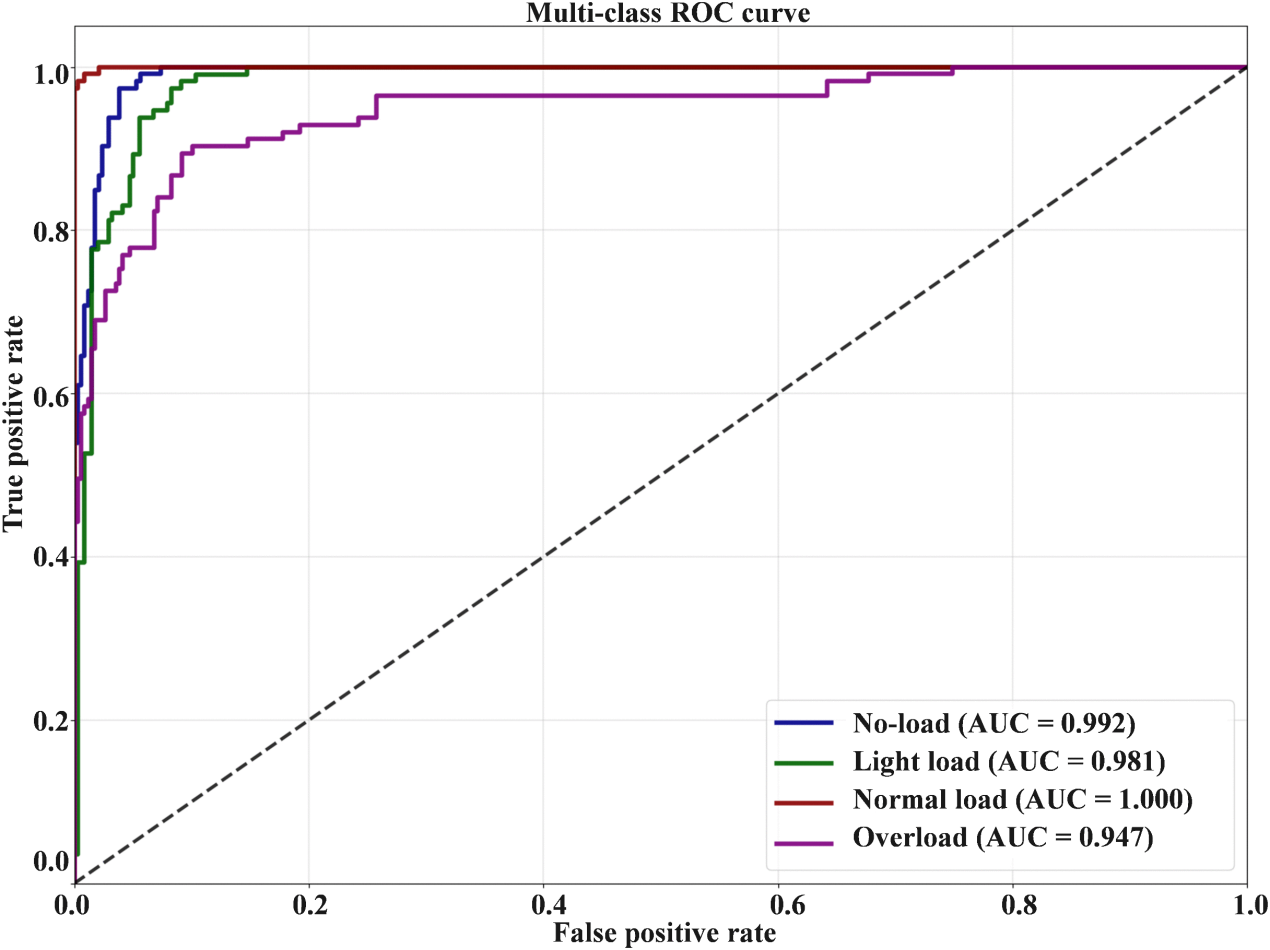
Multi-class ROC curves for load state identification. ROC curves are shown for no-load (blue), light load (green), normal load (red), and overload (purple), with AUC values of 0.992, 0.981, 1.000, and 0.947, respectively, demonstrating that the maximum-Lyapunov-exponent feature reliably distinguishes the four load states in real time.

The AUC values for no-load, light-load, normal-load, and overload were 0.992, 0.981, 1.000, and 0.947, respectively, providing quantitative evidence that the maximum-Lyapunov-exponent-based chaotic feature is reliable for real-time load identification under the tested conditions (Fig 9). In particular, normal load and no-load achieve near perfect discrimination, whereas light load and overload exhibit slightly lower but still excellent AUC values, confirming good separability among the four load conditions. The relatively lower AUC for overload suggests partial feature overlap with adjacent load states (especially normal load), which increases classification difficulty but remains acceptable for practical load differentiation.

### 3.8. Model comparison and computer experiments

#### 3.8.1. Purpose and experimental scheme of comparison.

The effectiveness of the proposed load identification method based on the chaotic dynamic characteristics of vibration-torque coupling is further validated through comparative experiments. To ensure the fairness and consistency of the comparison, all methods were tested on the same dataset under the same training and testing conditions. The identification performance was comprehensively evaluated using several commonly adopted metrics, including accuracy, precision, recall, F1 score, confusion matrix, and ROC curve.

The proposed method integrates vibration and torque signals and performs load identification through coupled dynamic modeling, Lyapunov exponent extraction, and multi-class SVM classification. Compared with conventional approaches, this method has stronger physical interpretability and better adaptability under small-sample conditions. Therefore, it is more suitable for the load identification of pepper harvesting drums under complex operating environments.

To evaluate its performance, the proposed method was compared with three commonly used load identification methods. All compared models were established using the same dataset and identical training/testing partition strategies, so that the differences in recognition performance could be attributed mainly to the identification methods themselves rather than to variations in the experimental setting. The evaluation indices, including overall accuracy, precision, recall, F1 score, and AUC, provide a comprehensive basis for comparing the ability of different models to distinguish various load states of the pepper harvesting drum. The detailed comparison results are shown in Table 3.

**Table 3:** Comparison of Model Performance.

Test No.	Accuracy (%)	Precision (%)	Recall (%)	F1 (%)	AUC
Method 1	80.20	78.45	81.30	79.75	0.912
Method 2	82.10	80.12	83.25	81.60	0.917
Method 3	85.35	84.22	85.95	85.08	0.952
Proposed Method	90.47	89.85	91.12	90.48	0.992

From Table 3 can be seen that the proposed method achieved the best performance among all compared models. Its overall accuracy reached 90.47%, while the precision, recall, and F1 score were 89.85%, 91.12%, and 90.48%, respectively. In addition, the AUC value reached 0.992, indicating that the proposed method has excellent classification capability and strong discriminative power for different load states. The improvement in classification performance is related to the stable parameter estimation obtained by the hybrid GA-Gauss-Newton procedure. The GA reduces the probability of local convergence by providing globally searched initial parameters, while Gauss-Newton refinement improves the fitting precision of the nonlinear coupled model. Therefore, the reconstructed vibration-torque dynamics are represented more accurately, and the extracted Lyapunov-based features become more discriminative for load-state classification. This explains why the proposed method achieved higher accuracy, precision, recall, F1 score, and AUC than the compared conventional methods. These results demonstrate that the vibration-torque coupling-based method outperforms conventional methods in terms of identification accuracy and overall robustness.

#### 3.8.2. Experimental results and analysis.

The comparative results further confirm the superiority of the proposed method in load-state recognition. As shown in Table 3, the proposed method outperformed the other three comparison models in all major evaluation metrics. This indicates that the combination of coupled dynamic modeling, Lyapunov exponent extraction, and multi-class SVM classification can effectively capture the intrinsic dynamic differences among different load states.

In terms of overall recognition performance, the proposed method achieved an accuracy of 90.47%, which was significantly higher than those of Method 1, Method 2, and Method 3. The improvements in precision, recall, and F1 score also demonstrate that the proposed method not only improves the overall classification accuracy but also maintains better balance among different categories. This suggests that the proposed method is more effective in reducing both false positives and false negatives during load-state identification.

With respect to discriminative ability, the AUC value of 0.992 indicates that the proposed method has strong classification reliability and excellent separability among different load categories. This result confirms that the extracted chaotic features can effectively represent the dynamic characteristics of the drum under different operating loads. Compared with conventional methods based on single-signal features or simple feature concatenation, the proposed method makes better use of the intrinsic coupling relationship between vibration and torque signals, thereby improving identification performance.

The results also suggest that the proposed method is particularly suitable for complex agricultural operating conditions, where load variations are highly nonlinear and strongly affected by external disturbances. Under such conditions, traditional methods are often limited by weak physical interpretability and insufficient sensitivity to subtle load changes, whereas the proposed method can provide a more robust and physically meaningful solution for load identification.

#### 3.8.3. Summary of advantages of the proposed method.

The proposed method exhibits several key advantages compared with traditional load identification techniques. High recognition accuracy is achieved, with an overall accuracy of 90.47% and superior AUC performance, particularly in differentiating between normal-load and no-load conditions. The method is rooted in the coupled dynamic modeling of vibration and torque, using Lyapunov exponents to provide a more physically interpretable approach to load identification. Traditional techniques often lack a robust physical foundation, a limitation addressed effectively by the proposed method.

High performance is maintained under small sample scenarios. This constitutes a significant advantage over deep learning methods, which typically require large datasets to achieve high precision. The proposed method delivers strong load identification capability. High training efficiency is another notable feature, enhancing practicality in scenarios where data availability or computational resources may be limited.

Substantial potential for engineering applications is inherent to the method. Successful implementation on the Raspberry Pi 4B has been achieved, demonstrating capability in real-time signal acquisition and load identification in on-site agricultural machinery environments.

Substantial potential for engineering applications is inherent to the method. Successful implementation on the Raspberry Pi 4B has been achieved, demonstrating capability in real-time signal acquisition and load identification in on-site agricultural machinery environments. As shown in Fig 9, the software system and experimental setup for the on-machine load identification of the pepper harvesting drum intuitively reflects the practical application effect of the proposed method in the actual working environment of agricultural machinery.

**Fig 10:**
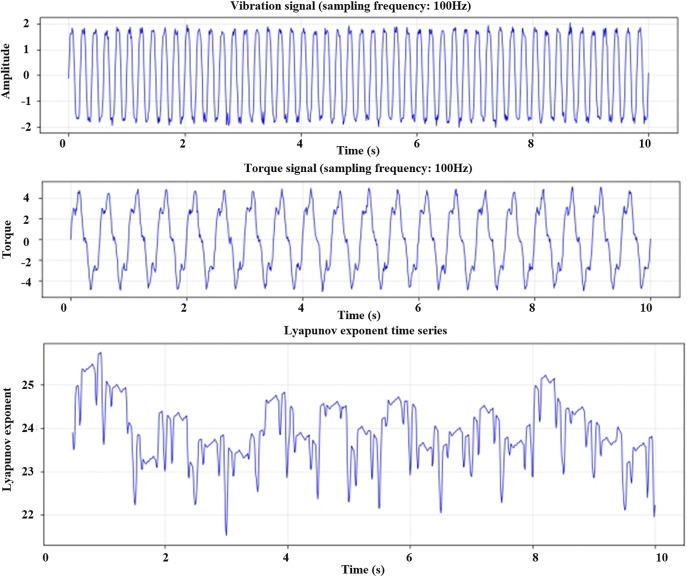
Experimental Study on the Load Identification Software System of Chili Harvester Drum. Time-series vibration (top) and torque (middle) signals, and Lyapunov exponent series (bottom), demonstrating the Raspberry Pi 4B-based system’s real-time load identification with high accuracy and practical applicability.

These experimental setups further validate the enormous engineering application potential of the proposed method, ensuring its applicability to real-world applications where load monitoring and adaptive control are crucial for improving harvesting efficiency and reducing operating costs (Fig 10).

## 4. Discussion

### 4.1. Model construction and data validation

By integrating chaos theory with nonlinear dynamical modeling, the proposed dual chaos framework establishes a mathematical representation capable of characterizing the complex operating behavior of the pepper harvesting drum. Drum vibration and torque signals were used as input variables, and phase space reconstruction and Lyapunov-exponent analysis were employed to extract load-sensitive chaotic features. Validation using measured operational data indicates that the proposed model can capture subtle dynamical differences across load changes. For instance, during transitions from light load to heavy load, the maximum Lyapunov exponent exhibits a characteristic trend of first increasing and then decreasing, which is consistent with the physical evolution from stable motion to a chaotic regime and then to a new stable state. In addition, the classification results (confusion matrix and ROC-AUC) demonstrate strong load-recognition capability across the four load states.

### 4.2. Comparative advantages over traditional methods

Compared with traditional load identification methods based on statistical analysis or single physical models, the dual chaos nonlinear dynamic model has significant advantages. Traditional methods often assume that the system operates in a linear or simply nonlinear state, making it difficult to accurately describe the drum’s dynamic characteristics under complex working conditions. In addition, compared with deep learning approaches, the proposed method has lower dependence on large-scale labeled datasets and provides stronger physical interpretability. Although deep learning models can automatically learn complex feature representations, their performance usually depends on sufficient training samples and may be affected by overfitting or limited generalization under small-sample and highly variable field conditions. By contrast, the proposed method extracts the maximum Lyapunov exponent from the vibration-torque coupled chaotic model as a load-sensitive feature, enabling effective load-state identification while maintaining a clear mechanistic explanation of the relationship between load variation and dynamic response. In contrast, relying on the sensitive dependence of chaotic systems on initial conditions and the ability of nonlinear dynamics to characterize the coupling effects of multiple factors, this model can more comprehensively reflect the impact of load changes on the drum system. Furthermore, the model maintains good generalization ability under small sample data conditions. It can achieve accurate identification of loads under different working conditions by learning from limited samples, which holds important practical value for industrial scenarios where data collection is limited. Comparative experiment results show that the proposed method outperforms conventional methods in all key evaluation metrics including accuracy, precision, recall, F1 score and AUC. This superiority is attributed to the full exploitation of the intrinsic coupling relationship between vibration and torque signals, and the extraction of physically interpretable chaotic features that are highly sensitive to load changes, which makes up for the defects of traditional methods.

### 4.3. Engineering application value

The proposed framework has practical value for load monitoring and optimal control of drum equipment. It has the potential to support online load identification and adaptive adjustment of operating parameters (e.g., feed rate regulation and drive power adaptation) according to load changes, thereby improving efficiency and reducing energy consumption. The method also provides a potential pathway for early fault warning, as abnormal loads often precede mechanical failures; continuous monitoring of load related dynamical features may help identify potential issues (e.g., bearing wear or poor gear meshing) in advance and support predictive maintenance. The successful implementation of the method on Raspberry Pi 4B realizes on-machine real-time signal acquisition and load identification, which verifies its feasibility in actual agricultural machinery field operations.

### 4.4. Model limitations

Although the proposed vibration-torque coupled chaotic dynamic model demonstrates good performance in drum load identification, several limitations remain and should be acknowledged.

First, the present experiments were mainly conducted on a dedicated test platform under controlled or field-like conditions. In practical field harvesting, spring-tooth drums are subjected to uncertain agricultural disturbances, including variations in plant architecture and fruit distribution density, uneven feeding, plant lodging, changes in fruit maturity and moisture content, soil-induced vibration caused by uneven ground, and impurity mixing such as leaves, stems, and soil clods. These factors may lead to time-varying model parameters and degrade load-identification accuracy. Therefore, the generalization ability of the proposed method under different pepper varieties, planting densities, soil conditions, and long-term continuous field operation still requires further validation.

Second, the proposed Lorenz-Rössler coupled model involves nonlinear coupling, numerical integration, parameter estimation, and Lyapunov-exponent calculation, which increases computational complexity compared with simple statistical feature-based methods. The main computational burden arises from solving the six-dimensional coupled dynamical system, performing GA-Gauss-Newton parameter estimation, and calculating the maximum Lyapunov exponent. Among these steps, GA-Gauss-Newton optimization is the most time-consuming, but it is mainly conducted during offline model calibration rather than repeated in each real-time identification window. During online load identification, the computation mainly includes signal preprocessing, state reconstruction, Lyapunov-exponent extraction, and SVM classification, so the real-time burden is relatively limited. Although the Raspberry Pi 4B implementation verified the feasibility of on-machine real-time identification, further model simplification, optimized numerical integration, sliding-window updating, and lightweight edge-computing implementation are still needed for high-frequency signal processing and stricter low-latency control applications.

Third, the method depends on the accuracy and reliability of torque and vibration sensors. Nevertheless, the relatively lower AUC of the overload state indicates that boundary discrimination under disturbed high-load conditions remains the main limitation. In addition, the real-time performance may still be affected by sensor noise, installation error, uneven feeding, crop-density variation, and soil-induced vibration. Future work will focus on adaptive parameter updating, robust signal preprocessing, sensor fault diagnosis, computational simplification, and multi-source feature fusion to improve overload-boundary discrimination and real-time robustness. Therefore, robust signal preprocessing and sensor fault diagnosis should be further considered in future applications.

Fourth, the comparative experiments mainly involved several conventional load identification methods. More advanced deep learning methods and hybrid physics-informed models should be included in future comparisons to further evaluate the relative advantages of the proposed method. In addition, the on-machine experiment was verified in a specific test environment, and the long-term stability and transferability of the method under actual large-area field harvesting still need further verification.

### 4.5. Future research directions

To overcome the above limitations and improve engineering applicability, future research can be pursued in the following directions:

(1) Adaptive parameter updating and hybrid frameworks combining the dual chaos model with recurrent architectures will be explored to improve adaptability while preserving physical interpretability. Comparisons with LSTM, CNN, Transformer-based models, and hybrid physics-informed models will also be conducted under a unified evaluation protocol using metrics such as RMSE, accuracy, F1-score, AUC, inference time, and model complexity.
(2) Efficiency-oriented implementation. Develop reduced order or simplified representations of the nonlinear coupling model, such as local linear approximation, reduced order modeling, or manifold learning, to decrease computational cost while retaining discriminative dynamic features. Edge computing deployment can also be investigated to enable on machine real time processing and fast response.
(3) Multi-source data fusion. Extend vibration-torque coupling by incorporating additional sensing modalities (e.g., temperature, acoustic signals, or motor current) and operational parameters (e.g., forward speed and drum rotational speed). Multi-source fusion is expected to strengthen robustness against complex interferences and improve boundary discrimination between adjacent load states.
(4) Validation across different pepper varieties, planting densities, plant architectures, fruit maturity levels, moisture contents, soil conditions, and disturbance scenarios will be conducted to evaluate robustness and generalization. Environmental disturbances such as uneven ground excitation, soil-induced vibration, plant lodging, and impurity interference will be considered to verify model transferability under complex field conditions. The proposed framework will also be extended to other agricultural rotating drum devices to assess its engineering applicability.

## 5. Conclusion

This study proposed a load-state identification method for pepper harvesting drums based on vibration-torque coupled chaotic dynamics. By integrating vibration and torque signals into a six-dimensional nonlinear coupled framework, the method provides a physically interpretable approach for drum load monitoring under complex harvesting disturbances.

(1) The proposed framework characterizes drum load through the nonlinear coupled evolution of vibration and torque, extending agricultural load identification from empirical feature classification to mechanism-consistent dynamic modeling. Combined with a multi-class SVM classifier, the Lyapunov-exponent-based feature achieved an average identification accuracy of 90.47%, with AUC values of 0.992, 0.981, 1.000, and 0.947 for no-load, light-load, normal-load, and overload states, respectively.
(2) The optimized operating parameters were 150 r/min drum rotational speed and 0.42 m/s forward speed, under which the picking rate reached 99.05% and the fruit damage rate was 2.35%. Comparative experiments and embedded implementation confirmed the engineering feasibility of the proposed framework.
(3) Despite these contributions, several limitations remain. Overload-boundary discrimination needs further improvement, computational complexity may constrain stricter low-latency applications, and transferability under broader crop varieties, planting densities, field disturbances, and other agricultural rotating drum devices requires systematic validation.

## Supporting information

S1 File The code used in this study. (ZIP)

S2 File The raw data of the experiments. (ZIP)
