# Correction: Load identification method for pepper harvesting drum based on dynamic chaotic characteristics of vibration-torque coupling

**DOI:** 10.1371/journal.pone.0355707

**Published:** 2026-08-07

**Authors:** Chen Wei, Jin Lei, Xinyan Qin, Yunshu Xiao, Zhi Wang, Shiguo Wang, Bin Li, Xiaohu Guo, Chengfu Wang

The affiliation for the second author is incorrect. Jin Lei is not affiliated with #2 but with #1: College of Mechanical and Electrical Engineering, Shihezi University, Shihezi, China.
